# SNP panels for the estimation of dairy breed proportion and parentage assignment in African crossbred dairy cattle

**DOI:** 10.1186/s12711-021-00615-4

**Published:** 2021-03-02

**Authors:** Netsanet Z. Gebrehiwot, Eva M. Strucken, Karen Marshall, Hassan Aliloo, John P. Gibson

**Affiliations:** 1 Centre for Genetic Analysis and Applications, School of Environmental and Rural Science, University of New England, Armidale, NSW 2351 Australia; 2 International Livestock Research Institute and Centre for Tropical Livestock Genetics and Health, Nairobi, Kenya

## Abstract

**Background:**

Understanding the relationship between genetic admixture and phenotypic performance is crucial for the optimization of crossbreeding programs. The use of small sets of informative ancestry markers can be a cost-effective option for the estimation of breed composition and for parentage assignment in situations where pedigree recording is difficult. The objectives of this study were to develop small single nucleotide polymorphism (SNP) panels that can accurately estimate the total dairy proportion and assign parentage in both West and East African crossbred dairy cows.

**Methods:**

Medium- and high-density SNP genotype data (Illumina BovineSNP50 and BovineHD Beadchip) for 4231 animals sampled from African crossbreds, African *Bos taurus*, European *Bos taurus, Bos indicus,* and African indigenous populations were used. For estimating breed composition, the absolute differences in allele frequency were calculated between pure ancestral breeds to identify SNPs with the highest discriminating power, and different combinations of SNPs weighted by ancestral origin were tested against estimates based on all available SNPs. For parentage assignment, informative SNPs were selected based on the highest minor allele frequency (MAF) in African crossbred populations assuming two Scenarios: (1) parents were selected among all the animals with known genotypes, and (2) parents were selected only among the animals known to be a parent of at least one progeny.

**Results:**

For the medium-density genotype data, SNPs selected for the largest differences in allele frequency between West African indigenous and European *Bos taurus* breeds performed best for most African crossbred populations and achieved a prediction accuracy (*r*^*2*^) for breed composition of 0.926 to 0.961 with 200 SNPs. For the high-density dataset, a panel with 70% of the SNPs selected on their largest difference in allele frequency between African and European *Bos taurus* performed best or very near best across all crossbred populations with *r*^*2*^ ranging from 0.978 to 0.984 with 200 SNPs. In all African crossbred populations, unambiguous parentage assignment was possible with ≥ 300 SNPs for the majority of the panels for Scenario 1 and ≥ 200 SNPs for Scenario 2.

**Conclusions:**

The identified low-cost SNP assays could overcome incomplete or inaccurate pedigree records in African smallholder systems and allow effective breeding decisions to produce progeny of desired breed composition.

**Supplementary Information:**

The online version contains supplementary material available at 10.1186/s12711-021-00615-4.

## Background

Africa has large populations of livestock with many different indigenous cattle populations [1]. In a recent synthesis of public domain and new genotype data, Gebrehiwot et al. [2] found that the sampled African indigenous cattle populations, with the exception of some pure African *Bos taurus* breeds found in West Africa, are admixtures between *Bos indicus* and African *Bos taurus*, and that West and Southern African populations showed a lower *Bos indicus* content than East African populations. Several African countries have introduced exotic cattle breeds over the last century with the objective of increasing the productivity of indigenous breeds [1]. These exotic breeds differ between countries, depending on the country’s history and connection to Europe. In East African countries, Holstein and Friesian were predominantly imported, but some countries such as Kenya and Tanzania also imported Ayrshire. In West African countries under French influence, Montbeliarde and Holstein–Friesian have been the favored exotic breeds [3–7].

Genetic evaluations of the indigenous breeds and the crossbreds have not been systematically performed mainly due to poor performance and pedigree recording [8]. Knowledge of the genetic relationships in populations is an important tool for evaluating the suitability and adaptability of animals to production environments, sorting animals into management groups, and estimating quantitative genetic parameters and breeding values [9]. Correct parentage assignment remains important for a successful breeding program, so that production performances can be associated with family relationships to improve estimates of breeding values [10, 11].

Conventionally, pedigree data have been used to determine the relationships between individuals, but pedigree information is rarely recorded in many developing countries [12, 13]. Molecular genetic markers, such as single nucleotide polymorphisms (SNPs), can be used for parentage assignment and pedigree reconstruction [14, 15], or to obtain estimates of breed composition in crossbred populations, using genotypes from reference purebred populations that may have contributed to the crossbred population. Studies of breed compositions in cattle have been mostly based on microsatellites [16, 17] or medium- to high-density SNP assays [2, 18, 19]. However, the major limitation to a wider use of molecular markers in developing countries is the cost of genotyping, and the expected value of the information gained by genotyping must exceed the cost of obtaining the genotypes [20].

Several studies have shown that a small set of SNPs, if accurately chosen, is sufficient to differentiate the genetic origin of breeds [15, 21]. In the case of the estimation of breed composition, Kumar et al. [22] showed that a subset of 470 informative SNPs could discriminate six Indian indigenous and European dairy breeds. Using a combination of principal component analysis (PCA) and random forest, Hulsegge et al. [23] identified 133 SNPs that allowed to differentiate local Dutch cattle breeds. The internationally recognized International Society for Animal Genetics (ISAG) SNP panel for parentage assignment in cattle originally had 100 SNPs [24] but was later expanded to 200 SNPs to provide more accurate assignments in a wider range of breeds [25]. Bertolini et al. [26] identified 96 informative SNPs to discriminate six cosmopolitan and Italian local cattle breeds. Fisher et al. [27] found that a 40-SNP panel (with a mean minor allele frequency (MAF of 0.35) could be sufficient to undertake parentage testing with high accuracy when combined with mating records and birth dates in New Zealand dairy herds.

Strucken et al. [15] used 735 k SNPs to develop small sub-sets of markers to estimate total dairy breed proportion and assign parentages in East African crossbred dairy cattle. The authors found that appropriately selected panels of 200 to 400 SNPs could be used to estimate the total dairy breed proportion with high accuracy (${r}^{2}$ >0.97). Similar sized informative SNP sub-sets that were selected based on their highest MAF in crossbred animals were able to assign parentages unequivocally. However, it is not known how well these small SNP assays will work for breed composition estimation or parentage assignments in crossbred populations in other parts of Africa where the indigenous genetic base and the exotic dairy breeds may differ from those in East African countries.

Here, we identified small subsets of SNPs that provide accurate estimates of total dairy breed proportion and parentage assignment across crossbred populations from East and West Africa with different indigenous and exotic ancestries. Then, we compared the estimation of breed proportion with small SNP panels selected from medium- and high-density SNP panels, and also tested the performance of the existing small SNP panels selected in East African crossbred populations for West Africa crossbred populations.

## Methods

### Animals

In total, 667 African indigenous cattle and 3334 indigenous cattle crossed to exotic dairy breeds were sampled from Senegal, Kenya, Uganda, Ethiopia, and Tanzania (Table 1). Data for East and West African countries were obtained from several public-domain databases plus projects run by the International Livestock Research Institute (ILRI) and collaborators (Marshall et al. [28], Marshall et al. [29], Ema et al. [30]), the Centre for Tropical Livestock Genetics and Health (CTLGH), and the Dairy Genetics East Africa project (DGEA, [15]).

**Table 1:** Animal populations, numbers, and sources

Breed	Breed group	Geographical location	Origin/country	Number of animals	Array (Illumina)	Genotype source
Reference samples						
Ayrshire	EuB.t	Canada	Canada	20	BovineHD	Canadian Dairy Network
Friesian	EuB.t	European	UK	25	BovineHD	SRUC
Guernsey	EuB.t	USA and UK	USA and UK	20	BovineHD	Bovine HapMap et al. [32]
Holstein	EuB.t	USA and NZ	USA and NZ	20	BovineHD	Bovine HapMap consortium et al. [32]
Jersey	EuB.t	USA and NZ	USA and NZ	20	BovineHD	Bovine HapMap consortium et al. [32]
Pooled *Bos indicus*	B.i	Indian	India	105	BovineHD	Strucken et al.^a^
Baoule	AfB.t	West African	Burkina Faso	9	BovineHD	GRRFAC
N’Dama	AfB.t	West African	Guinea	20	BovineHD	Bovine HapMap consortium et al. [32]
N’Dama	AfB.t	West African	Senegal	7	BovineHD	GRRFAC
Montbeliarde	EuB.t	European	France	20	BovineSNP50	Decker et al. [18]
N’Dama1	AfB.t	West African	Cote d’Ivoire	14	BovineSNP50	Decker et al. [18]
Baoule	AfB.t	West African	Burkina Faso	20	BovineSNP50	Decker et al. [18]
Lagune	AfB.t	West African	Benin	20	BovineSNP50	Decker et al. [18]
Somba	AfB.t	West African	Togo	20	BovineSNP50	Decker et al. [18]
Target populations						
Sheko1	Sanga	East African	Ethiopia	18	BovineHD	Bovine HapMap consortium et al. [32]
Ankole	Sanga	East African	Uganda	35	BovineHD	DGEA [15]
SEAZ	zebu	East African	Kenya	21	BovineHD	DGEA [15]
Danakil-Harar	zebu	East African	Ethiopia	30	BovineHD	DGEA [15]
Begait-Barka	zebu	East African	Ethiopia	27	BovineHD	DGEA [15]
Boran	zebu	East African	Ethiopia	28	BovineHD	DGEA [15]
Boran	zebu	East African	Kenya	28	BovineHD	DGEA [15]
Fogera	zebu	East African	Ethiopia	28	BovineHD	DGEA [15]
Iringa-Red	zebu	East African	Tanzania	11	BovineHD	DGEA [15]
Singida-White	zebu	East African	Tanzania	22	BovineHD	DGEA [15]
Central Highland	zebu	East African	Ethiopia	9	BovineHD	DGEA [15]
Kenyan crossbred	Crossbred	East African	Kenya	1378	BovineHD	DGEA [15]
Uganda crossbred	Crossbred	East African	Uganda	555	BovineHD	DGEA [15]
Ethiopia crossbred	Crossbred	East African	Ethiopia	545	BovineHD	DGEA [15]
Tanzania crossbred	Crossbred	East African	Tanzania	462	BovineHD	DGEA [15]
Sheko	Sanga	East African	Ethiopia	17	BovineSNP50	Decker et al. [18]
Madagascar-zebu	zebu	Madagascar	Madagascar	20	BovineSNP50	Decker et al. [18]
Lagunaire	AfB.t	West African	West Africa	5	BovineHD	Bovine HapMap consortium et al. [32]
Senegal crossbreed	Crossbred	West African	Senegal	141	BovineHD	CTLGH
N’Dama2	AfB.t	West African	Southeast Burkina Faso	14	BovineSNP50	Decker et al. [18]
N’Dama3	AfB.t	West African	Southwest Burkina Faso	17	BovineSNP50	Decker et al. [18]
Djakore	zebu	West African	Senegal	7	BovineSNP50	Marshall et al. [28]
Gobra	zebu	West African	Senegal	118	BovineSNP50	Marshall et al. [28]
Maure	zebu	West African	Senegal	12	BovineSNP50	Marshall et al. [28]
Gobra x Maure	zebu	West African	Senegal	10	BovineSNP50	Marshall et al. [28]
Bororo	zebu	West African	Chad	20	BovineSNP50	Decker et al. [18]
Fulani	zebu	West African	Benin	20	BovineSNP50	Decker et al. [18]
Kuri	AfB.t	West African	Chad	20	BovineSNP50	Decker et al. [18]
Borgou	zebu	West African	Benin	20	BovineSNP50	Decker et al. [18]
Senegal crossbreed	Crossbred	West African	Senegal	253	BovineSNP50	Marshall et al. [28]
Total				4231		

EuB.t = European *Bos taurus*, AfB.t = African *Bos taurus,* B.i = *Bos indicus,* USA = United States of America, UK = United Kingdom, NZ = New Zealand. All the indigenous and crossbred populations were used for total dairy proportion estimation, while only crossbreds were used for the parentage assignment study

^a^personal communication: Strucken EM, Gebrehiwot, NZ, Swaminathan M, Joshi S, Al kalaldeh M, and JP Gibson. Genetic diversity and effective population sizes of thirteen Indian cattle breeds

Purebred reference breeds were five African *Bos taurus* samples (N’Dama, N’Dama1, Lagune, Baoule, and Somba), a pooled Indian *Bos indicus* sample (N = 105), and six European *Bos taurus* dairy breeds (Guernsey, Holstein, Jersey, Ayrshire, Friesian, and Montbeliarde, N = 125) as described in detail by Gebrehiwot et al. [2], with the origin of samples in Table 1. The pooled *Bos indicus* sample included 12 *Bos indicus* breeds from India, selected from 525 indigenous samples such that within-breed relationships were minimal [31]. A pooled sample was used for *Bos indicus* because Strucken et al. [personal communication: Strucken EM, Gebrehiwot, NZ, Swaminathan M, Joshi S, Al kalaldeh M, and JP Gibson: Genetic diversity and effective population sizes of thirteen Indian cattle breeds] found remarkably little genetic diversity between *Bos indicus* breeds, and all *Bos indicus* breeds have extremely different allele frequencies compared to *Bos taurus* breeds.

### Genotypes and quality control

The samples were genotyped on either the Illumina BovineSNP50v2 BeadChip array (Illumina Inc., San Diego, USA) comprising 54,609 SNPs or the Illumina BovineHD Beadchip (Illumina Inc., San Diego, USA) comprising 777,962 SNPs (Table 1). Data obtained from the Bovine HapMap Consortium [32], the Canadian Dairy Network (CDN), and the 50 k data from Decker et al. [18] were obtained post-quality control. Genotypes of the DGEA and Scotland’s Rural College (SRUC) data were filtered using the R pipeline from *SNPQC* [33], which retained the SNPs that had a median GC score greater than 0.6 and a call rate higher than 90%. The data from Senegal’s smallholder farms [28] were processed for quality control using the GenABEL package [34] in R [35], which retained SNPs and animals with call rates higher than 90%. A GC score was not available for this dataset. Data from CTLGH were quality-controlled, using a GC score greater than 0.6 and a call rate higher than 0.90%. Only autosomal SNPs were included in this study.

Two datasets were used in our analyses: (1) all available data were merged keeping only common SNPs across all datasets, which resulted in a subset of 38,214 SNPs; and (2) only the datasets from HD assays were merged, which resulted in a subset of 712,775 SNPs.

### Estimation of breed composition

A maximum likelihood model, as implemented in the software ADMIXTURE 1.23 [36], was used to estimate breed proportions of the crossbred animals in a supervised analysis. Due to the availability of different breeds for the two datasets described above, different numbers of reference breeds were used. A baseline of ‘*true’* breed proportions was established with *K* = 11 for the 38 k dataset and *K* = 7 for the 713 k dataset. The ancestral populations for the 38 k dataset (*K* = 11) were N’Dama, Lagune, Baoule, Somba, N = 20 each, and N’Dama1 (N = 14) as African taurine reference breeds; Ayshire, Guernsey, Holstein, Jersey, Montbeliarde, N = 20 each, and Friesian (N = 25) as European dairy reference breeds; and the pooled *Bos indicus* reference population (N = 105). For the 713 k dataset (*K* = 7), the ancestral populations were a pooled African *Bos taurus* population (N = 36) comprising both N’Dama samples and the Baoule, the pooled *Bos indicus* population (N = 105), and the European *Bos taurus* Ayshire, Guernsey, Holstein, Jersey, N = 20, each, and Friesian (N = 25). The reference breeds were chosen based on known crossbreeding history in the African countries where samples were collected, and based on previous studies, which had checked breed purity and suitability for analysis in African populations [2, 15, 37]. Pooling of reference breeds was only performed when little genetic variation was found between breeds according to Gebrehiwot et al. [2], who used the same data.

Estimating individual breed proportions even with large numbers of markers has been shown to be problematic, however, estimates of total dairy proportions were very robust to changes in admixture models [37]. Therefore, we focussed on the total dairy proportion in crossbred animals defined as the sum of the estimated breed proportions of all ancestral European dairy breeds.

The accuracy of predicting total dairy proportion with small subsets of SNPs was estimated as the coefficient of determination (${r}^{2}$) between the estimates from small SNP panels and the *’true’* estimate from either the 38 k or 713 k datasets.

The Pearson correlation coefficient ($r$) was calculated as:

$$r = \frac{{\sum \left( {{xi} - \tt \overline{x}} \right)\left( {{yi} - \tt \overline{y}{ }} \right)}}{{\sqrt {\sum \left( {{xi} - \tt \overline{x}} \right)^{2} \sum \left( {{yi} - \tt \overline{y}} \right)^{2} } }},$$

where $xi$ and $yi$ are the total dairy proportion of an individual estimated with a small subset and *’true’* estimate, respectively, and $\tt \overline{x}$ and $\tt \overline{y}$ are the mean of the total dairy proportion estimated with a small subset and *’true’* estimate, respectively.

### Selection of small SNP panels for estimation of breed proportion

To find the most informative markers for the estimation of breed composition, the absolute difference in allele frequency between two populations representing the ancestral breeds of the crossbred cattle was calculated. The absolute difference in allele frequency for a biallelic marker was calculated as | pA_i_—pA_j_ |, where pA_i_ and pA_j_ are the frequencies of allele *A* in the i^th^ and j^th^ population, respectively. Population-specific allele frequencies were calculated within African *Bos taurus*, European *Bos taurus*, *Bos indicus,* as well as for different groups of African indigenous populations as described later. These ancestral populations were chosen based on results from admixture and principal components analyses, which confirmed that African crossbred populations are crosses between European dairy breeds and local indigenous populations [2]. The indigenous populations themselves are old, probably ancient hybrids between *Bos indicus* and African *Bos taurus* [2, 15, 19]. To produce panels that allowed the selection of markers based on all three ancestral groups (European *Bos taurus*, African *Bos taurus*, *Bos indicus*), weighted panels were created as follows.

In a first approach (i), the absolute differences in allele frequency were obtained between African and European *Bos taurus* (AFT*vs*EUT), and between *Bos indicus* and European *Bos taurus* (IND*vs*EUT). Panels were then created by selecting 10 to 90% of SNPs in 10% increments, with the largest absolute difference in allele frequency between AFT*vs*EUT, and the remainder from the largest absolute difference in allele frequency between IND*vs*EUT.

In a second approach (ii), allele frequencies in African *Bos taurus* and *Bos indicus* reference populations were combined with weightings ranging from 0.1:0.9 to 0.9:0.1 in increments of 0.1, to create a hypothetical population AFT-BI. The absolute differences in allele frequency were then calculated between European *Bos taurus* and AFT-BI, and small SNP panels were selected based on the largest absolute differences in allele frequency.

Once the absolute differences in allele frequency were calculated and SNPs were sorted by the largest difference, datasets were pruned to minimize the selection of SNPs with low information content due to linkage disequilibrium (LD) with previously selected SNPs. To avoid ascertainment bias in the selection of SNPs, we performed a population independent pruning approach based on the physical distance between markers rather than the observed LD in the crossbred populations. First, we applied a minimum distance of one Mb, as is often used based on the assumption that there is minimal LD across this genomic distance. With this pruning criterion, we observed that there was a very distinct clustering across the genome of the SNPs that had large differences in allele frequency, and that clustering caused a reduction of accuracies for the estimation of breed proportion. An improved criterion for our selection of SNPs was to increase the physical distance to 3.5 Mb; however, this only allowed selection of less than 500 SNPs that had large differences in allele frequency. Thus, a stepwise pruning method was applied [personal communication: Strucken EM, Swaminathan M and JP Gibson: Small SNP panels for breed proportion estimation in Indian crossbred Dairy cattle], where the first 100 SNPs had a minimum distance between SNPs of 3.5 Mb, the next 200 SNPs had a minimum distance of 3 Mb, and additional SNPs had a minimum distance of 1.25 Mb. This provided a higher accuracy compared to a static distance.

Subsets of pruned 100, 200, 300, 400, 500, 1000, and 1500 SNP panels were selected based on approaches (i) and (ii) described earlier.

We further separated the African indigenous animals present in the 38 k dataset into several groups based on admixture and PCA results from Gebrehiwot et al. [2] who studied the same dataset: (1) all African indigenous breeds together, (2) a zebu breed group, (3) a Sanga breed group, (4) all West African indigenous animals including African *Bos taurus* reference breeds, (5) indigenous breeds that have less than 40% African *Bos taurus* ancestry, and (6) indigenous breeds that have more than 40% African *Bos taurus* ancestry but excluding the pure African *Bos taurus* reference breeds. Absolute differences in allele frequency were calculated between European *Bos taurus* and each of these six African indigenous populations (AllIndig*vs*EUT, zebu*vs*EUT, Sanga*vs*EUT, AllWestind*vs*EUT, < Indig40%AFT*vs*EUT, and > Indig40%AFT*vs*EUT, respectively). Estimates of total dairy breed proportions from selected small SNP panels zebu*vs*EUT, Sanga*v*sEUT, and < Indig40%AFT*vs*EUT are not further discussed here because they performed comparatively poorly in all scenarios.

The two approaches for obtaining the largest absolute differences in allele frequency were tested when SNPs were sampled from both the 38 k and 713 k datasets. Higher ${r}^{2}$ were obtained between the selected panels and the 38 k dataset using the first approach, while higher ${r}^{2}$ were achieved with the second approach for the 713 k dataset. Thus, results are presented only for the first approach for SNP panels from the 38 k dataset and the second approach for SNP panels from the 713 k dataset.

### Parentage assignment

The number of opposing homozygotes (*opH*) between all pairs of individuals from all African crossbreds was calculated from the 38 k dataset. Apart from genotyping errors, a parent and its offspring cannot have opposing homozygote genotypes for a given SNP. According to Strucken et al. [38], parent–offspring pairs can be assigned based on a maximum number of Mendelian inconsistencies due to genotyping errors (typically about 1%). Across all crossbreds (N = 3193), 301 parent–offspring pairs were identified which included 26 parents with two offspring each, and two parents with three offspring each. In crossbreds from East Africa (N = 2940), 262 parent–offspring pairs including 20 parents with two offspring each, and one parent with three offspring were identified. In crossbreds from Kenya-Ethiopia-Tanzania together (N = 2385), 199 parent–offspring pairs including 13 parents with two offspring each. Crossbreds from Uganda (N = 555), included 63 parent–offspring pairs with five parents with two offspring each; and crossbreds from Senegal (N = 253), included 39 parent–offspring pairs covering five parents with two, and one parent with three offspring. These reconstructed parent–offspring pairs were based on all available markers and used as the baseline to test parentage assignments with small SNP panels.

### SNP panels selection for parentage assignment

MAF was calculated for each SNP in populations consisting of all crossbred animals together (MAF-xbreds); all East African crossbreds (MAF-East); and Senegalese crossbreds (MAF-Senegal). Then, we calculated MAF across the crossbreds from Kenya, Ethiopia, and Tanzania, since they share a common zebu ancestry (MAF-KenEthiTanz), and for the Ugandan crossbreds, which have a Sanga ancestry (MAF-Uganda) [2].

Small SNP panels were selected based on the highest MAF in each of the crossbred groups because SNPs with high MAF generate the highest frequency of opposing homozygotes between unrelated individuals. Similar to the selection of SNPs for the estimation of breed proportion, markers were sorted by highest MAF and then pruned to achieve a minimum distance of one Mb. Here, we did not observe a clustering of SNPs with this minimal physical distance. The pruned subsets are denoted as MAF-Pruned-xbreds, MAF-Pruned-East, MAF-Pruned-Senegal, MAF-Pruned-KenEthiTanz, and MAF-Pruned-Uganda.

Parentage assignment was tested in two scenarios: (1) in which every animal is considered as a possible parent of every other animal that has been genotyped; and (2) in which animals can be grouped into those that are parents *versus* those that are progeny, and putative parents are selected only among the animals in the parent groups. The power of the small SNP panels selected from 38 k SNPs was evaluated using the separation value ($sv$, [38, 39]). The $sv$ is data-dependent, and exact values depend on the number of SNPs in the assay and their allele frequencies in the population under test [38]. However, when all conditions remain constant, the best panel for parentage assignment is the panel that has the highest positive $sv$*.* The $sv$ was calculated as:

$$sv = \min \left( {FR} \right) - {\text{max}}\left( {TR} \right),$$

where $FR$ is the number of opposing homozygotes between false (unrelated) parent–offspring relationship, and $TR$ is the number of opposing homozygotes in true parent–offspring relationship.

The power of assignment ($Pa$) and the power of exclusion ($Pe$) were calculated to identify the best small SNP panels in the two scenarios, allowing a frequency of opposing homozygotes in true parent–offspring pairs of 1%:

$$Pa=\frac{number\,of\,correct\,parent\,assignments}{\text{total number of parents}},$$

$$Pe=1-\frac{number\,of\,incorrect\,parent\,assignments}{\text{total number of parents}},$$

where the denominator is the number of parents identified as true based on the 38 k dataset [40, 41]. A parent–offspring assignment was made whenever the number of opposing homozygotes was less than 1% of the number of markers in the assay. Setting such a threshold for identifying parent–offspring pairs was previously used [38, 42, 43] as a compromise to minimize false rejection or false assignment of parentages when the number of SNPs is small.

### Performance of reference panels

We investigated the accuracy in Senegal crossbreds of the SNPs that were selected for the estimation of total dairy breed proportion and parentage assignment in East African crossbred cattle by Strucken et al. [15] (“reference panels”). This was used to determine the accuracy of the reference panels in East *versus* West Africa and to compare the SNP selection methods used by Strucken et al. [15] with the methods in our study. The method of calculating the largest absolute differences in allele frequency between populations, as described in Strucken et al. [15], is the same as our second approach with a 0.5:0.5 weighting between African *Bos taurus* and *Bos indicus* allele frequencies.

## Results and discussion

### Distribution of allele frequencies

**Fig. 1:**
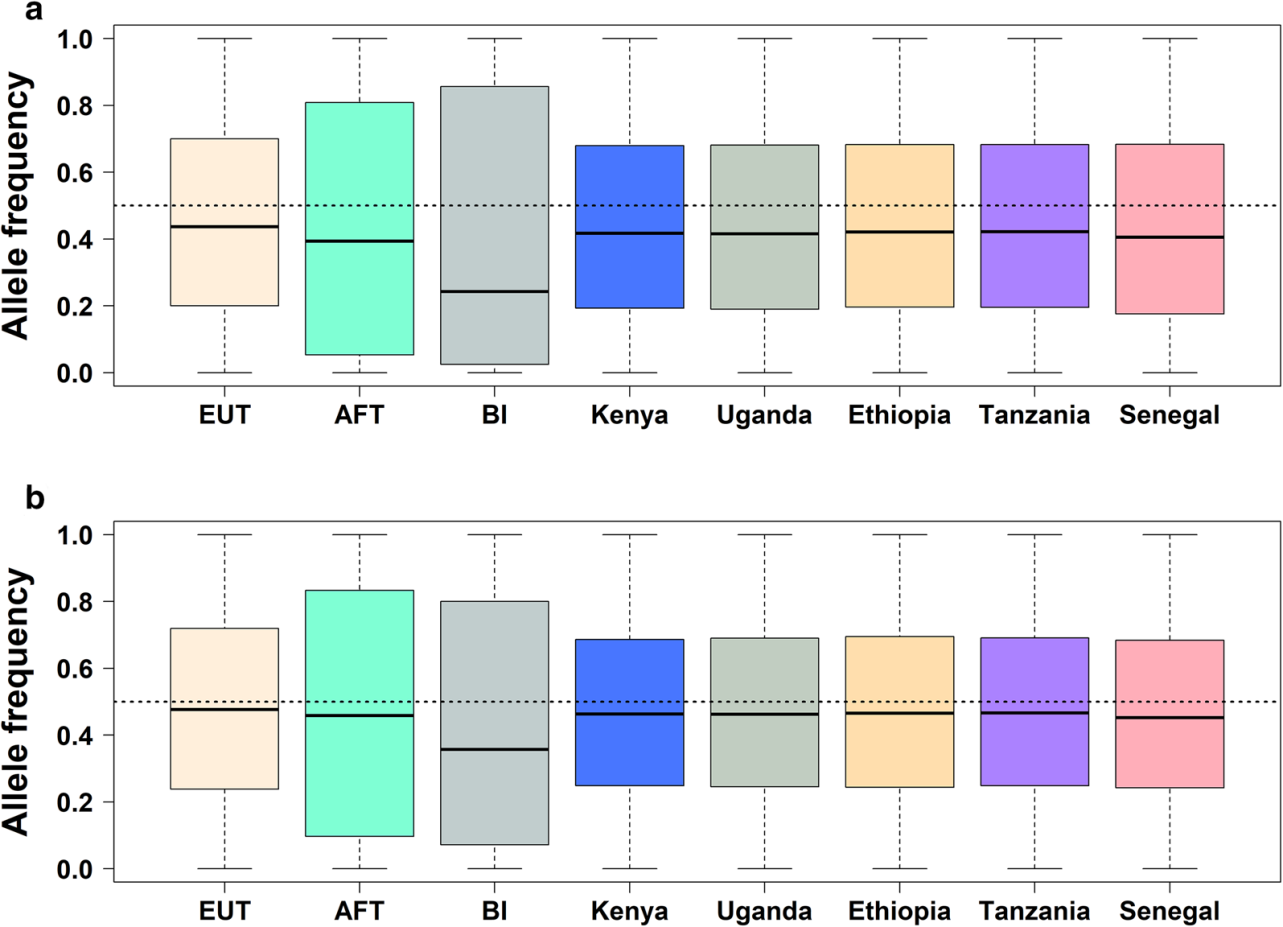
Boxplot of the observed allele frequencies in European *Bos taurus* (EUT), African *Bos taurus* (AFT), *Bos indicus* (BI), and African crossbred populations for **a** 38,214 SNPs **b** 712,775 SNPs

The distribution of observed allele frequencies of the 38 k and 713 k SNP datasets in ancestral and crossbred populations are shown in Fig. 1. In both datasets, the allele frequencies for African *Bos taurus* and *Bos indicus* showed a larger interquartile range than European *Bos taurus* and the crossbred animals, which indicates a higher dispersion of observed allele frequencies. The average allele frequency was biased away from 0.5 for all breeds, especially in the 38 k dataset (Fig. 1a). The *Bos indicus* reference showed the strongest deviation. This has been observed in previous studies [15], but the cause is unknown.

### Estimation of breed proportions using the 38 k and 713 k SNPs datasets

**Fig. 2:**
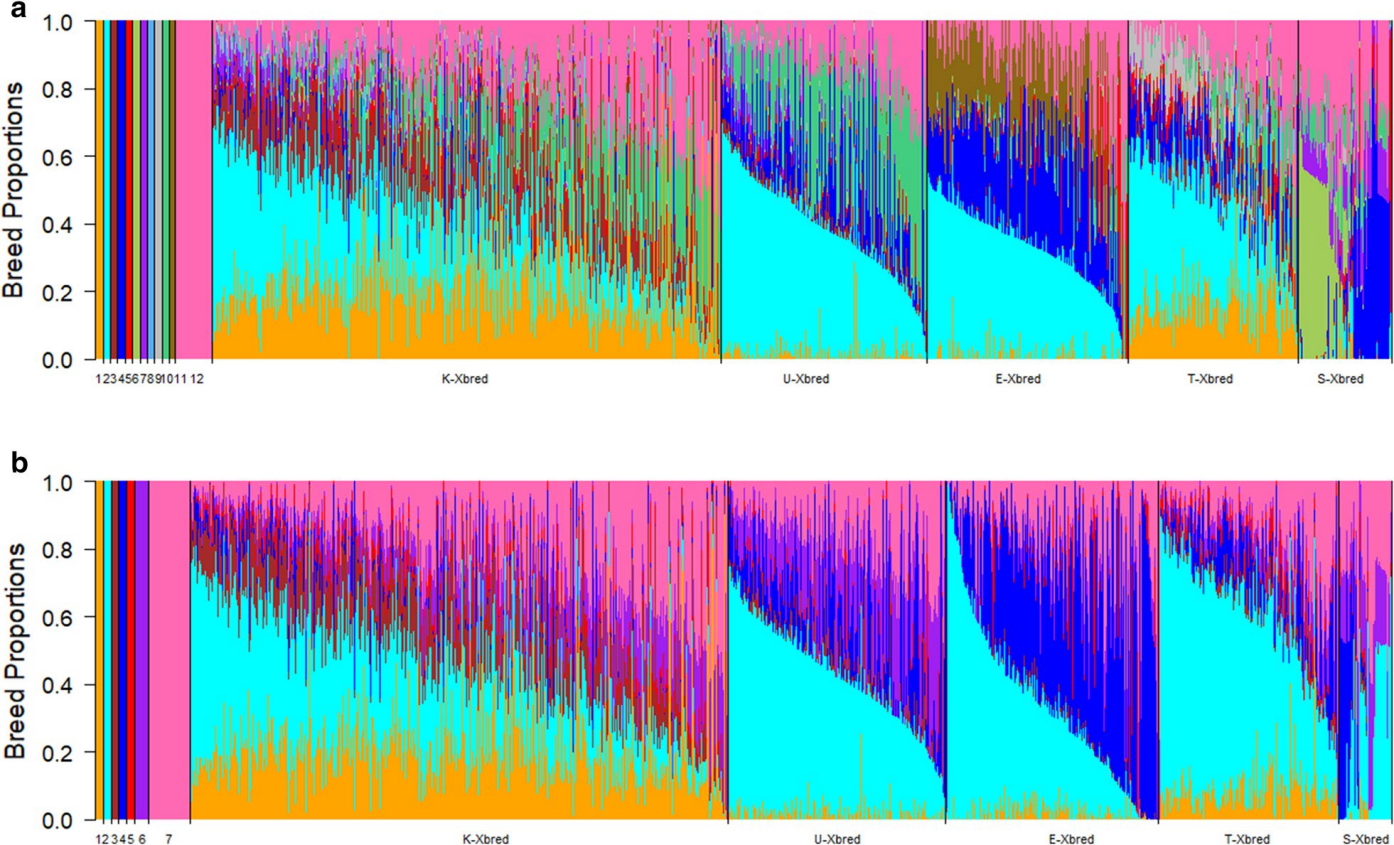
Breed proportion of crossbred cattle using **a** 38 k SNPs and **b** 713 k SNPs

Estimates of breed proportions using the 38 k and 713 k SNP datasets are presented in Fig. 2. Using the 38 k dataset (Fig. 2a), crossbred animals from Kenya, Ugandan, Ethiopian, Tanzanian and Senegal showed an average dairy proportion of 66% (± 0.199), 58% (± 0.190), 68% (± 0.168), 69% (± 0.169), and 51% (± 0.179), respectively. The average total dairy proportion using the 713 k dataset (Fig. 2b) was 71% (± 0.214), 65% (± 0.208), 79% (± 0.205), 79% (± 0.182), and 49% (± 0.197), respectively.

### Performance of small SNP panels for the estimation of total dairy breed proportion

#### Small SNP panels selected from the 38 k dataset

**Fig. 3:**
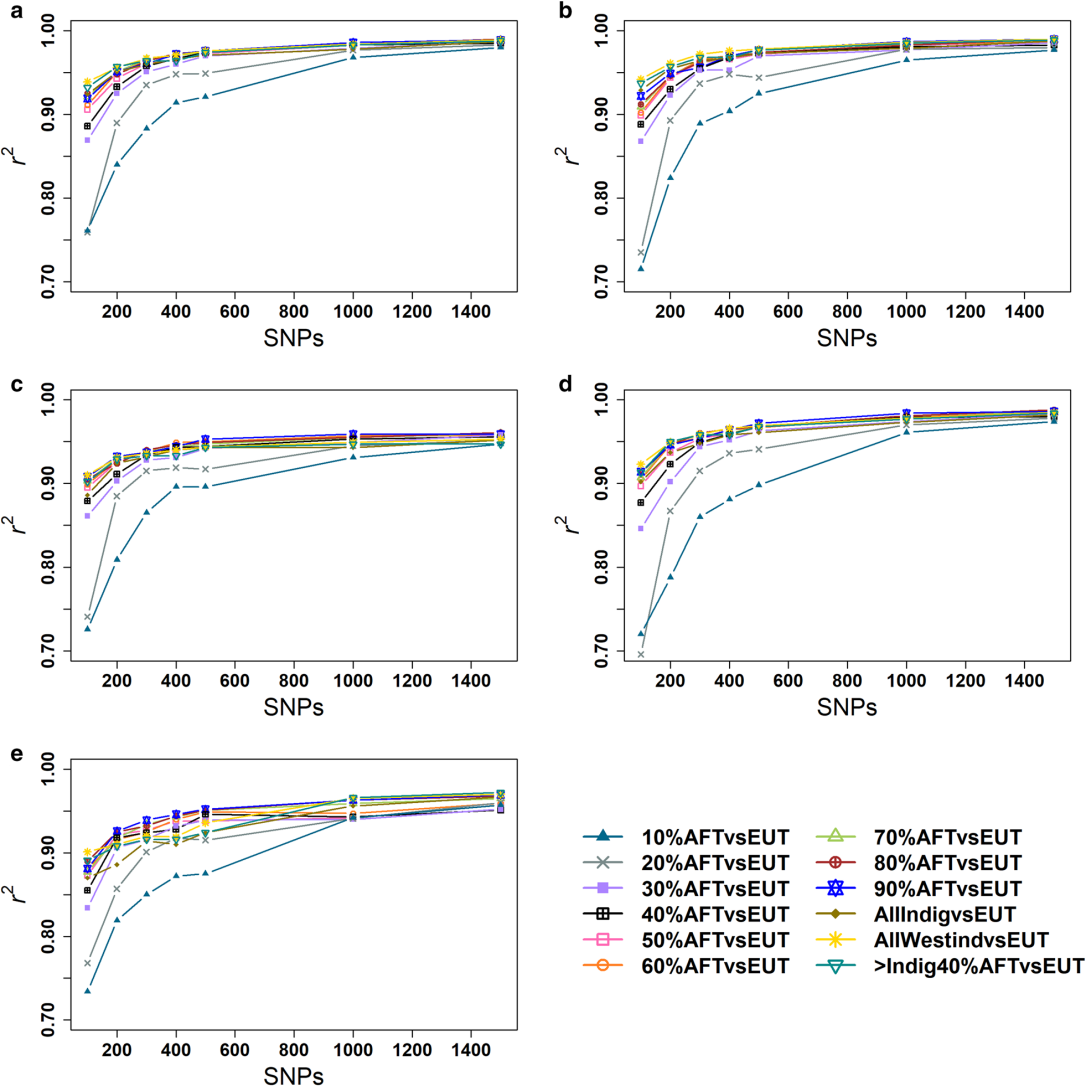
Accuracy (${r}^{2}$) of estimation of dairy breed proportion in crossbreds for panels selected from 38 k SNPs in **a** Kenya **b** Uganda **c** Ethiopia **d** Tanzania **e** Senegal

Estimates of total dairy breed proportion from selected small SNP panels were compared to those obtained from the full 38 k SNP set in different countries, and the accuracy of prediction was assessed using the coefficient of determination ${r}^{2}$. All small SNP panels showed a substantial increase in ${r}^{2}$ from 100 to 500 SNPs in all countries (Fig. 3). Increasing the number of SNPs beyond 500 resulted in smaller improvements in accuracy, which is consistent with the results of Strucken et al. [15]. Panels that had a higher proportion of markers selected to distinguish African from European *Bos taurus* proportions performed better compared to panels with more markers selected to distinguish *Bos indicus* from European *Bos taurus* populations. Panels with markers selected to distinguish African indigenous breed groups from European *Bos taurus* performed particularly well in East African crossbreds.

In Kenyan crossbreds, the accuracy of the dairy breed proportion estimated with the AllWestind*vs*EUT panel was higher than that with the other panels for 100, 300 and 500 SNPs with ${r}^{2}$ values equal to 0.939, 0.967, and 0.976, respectively, while the > Indig40%AFT*vs*EUT and 80%AFT*vs*EUT were the best panels with 200 (${r}^{2}$ = 0.957) and 400 (${r}^{2}$ = 0.973) SNPs (Fig. 3a). In Ugandan crossbreds, the AllWestind*vs*EUT panel achieved the highest accuracy compared to the other panels for 100 to 500 SNPs, with ${r}^{2}$ values ranging from 0.942 to 0.978 (Fig. 3b). In Ethiopian crossbreds, the AllWestind*vs*EUT, 80%AFT*vs*EUT and 60%AFT*vs*EUT performed better than the other panels with 100 (${r}^{2}$ = 0.910), 300 (${r}^{2}$ = 0.940) and 400 SNPs (${r}^{2}$ = 0.949), respectively, whereas the 90%AFT*vs*EUT was the best performing panel with 200 (${r}^{2}$ = 0.932) and 500 (${r}^{2}$ = 0.953) SNPs (Fig. 3c). In Tanzanian crossbreds, the AllWestind*vs*EUT was the best performing panel with 100 and 400 SNPs (${r}^{2}$ = 0.923 and 0.966). The > Indig40%AFT*vs*EUT and 80%AFT*vs*EUT and 90%AFT*vs*EUT were the best performing panels with 200 (${r}^{2}$ = 0.950), 300 (${r}^{2}$ = 0.960), and 500 (${r}^{2}$ = 0.972) SNPs, respectively (Fig. 3d). In Senegalese crossbreds, the AllWestind*vs*EUT was the best performing panel with 100 (${r}^{2}$ = 0.901) SNPs, whereas 90%AFT*vs*EUT was the best panel with 200 to 500 SNPs, with ${r}^{2}$ values ranging from 0.926 to 0.952. Except for the 100-SNP panel, the AllWestind*vs*EUT panel performed poorly compared to most of the other panels (Fig. 3e).

The AllWestind*vs*EUT panel performed best in most crossbred populations for most panel sizes. The differences in accuracy between the AllWestind*vs*EUT panel and the best performing panels for each panel size across different crossbred populations are in Table 2. The performance of the AllWestind*vs*EUT panel was worst for the Senegalese crossbreds where it provided a 0.001 to 0.026 lower accuracy than the best panel.

**Table 2:** Difference in ${r}^{2}$ between the AllWestind*vs*EUT and the best-performing SNP panels for dairy breed proportion prediction selected from 38 k SNPs

#SNPs	Kenya	Uganda	Ethiopia	Tanzania	Senegal
100	0.000	0.000	0.000	0.000	0.000
200	0.000	0.000	− 0.002	− 0.002	− 0.016
300	0.000	0.000	− 0.004	− 0.001	− 0.019
400	− 0.001	0.000	− 0.009	0.000	− 0.026
500	0.000	0.000	− 0.010	− 0.003	− 0.016
1000	− 0.002	0.000	− 0.009	− 0.006	− 0.001
1500	0.000	0.000	− 0.008	− 0.003	− 0.002

### Small SNP panels selected from the 713 k dataset

**Fig. 4:**
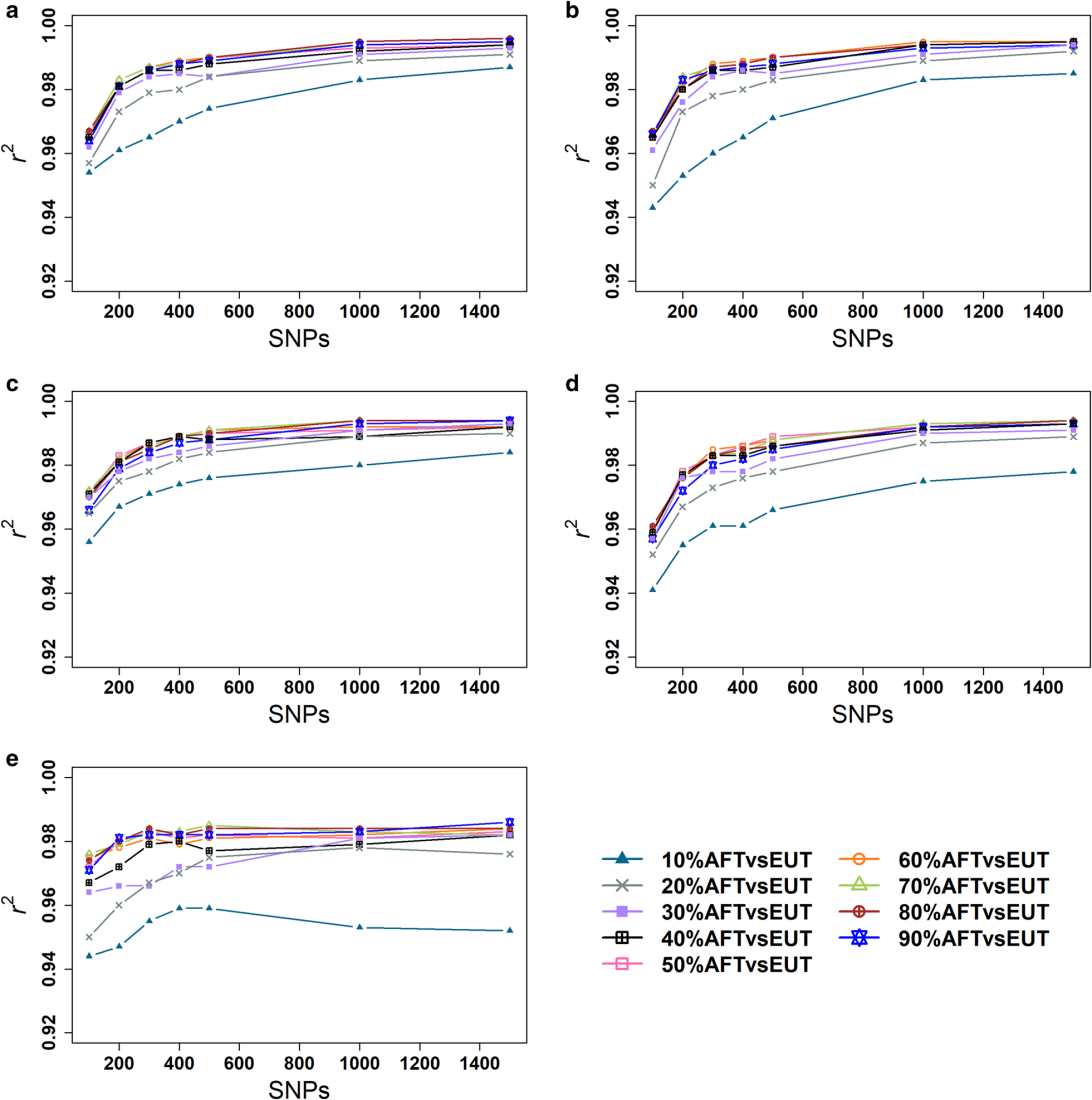
Accuracy (${r}^{2}$) of estimates of dairy breed proportion in crossbreds for panels selected from 713 k SNP in **a** Kenya **b** Uganda **c** Ethiopia **d** Tanzania **e** Senegal

Figure 4 presents the accuracy of estimates of dairy breed proportion for the small SNP panels selected from the 713 k SNP dataset. Similar to the results obtained with the 38 k dataset, the best performing panels always had a higher weighting on African *Bos taurus versus* European *Bos taurus* than on *Bos indicus versus* European *Bos taurus* allele frequency differences. As for the panels selected from the 38 k SNPs, the gains in accuracy were asymptotic with the number of SNPs, in this case, showing only small gains in accuracy with more than 300 SNPs.

In Kenyan crossbreds, the accuracy of dairy breed proportion estimation with the 70%AFT*vs*EUT panel was higher than with the other panels for 100 to 300 and 500 SNPs with ${r}^{2}$ values ranging from 0.967 to 0.987 and 0.990, respectively, whereas the 60%AFT*vs*EUT was the best performing panel for 400 SNPs (${r}^{2}$ = 0.989) (Fig. 4a). In Ugandan crossbreds, the 80%AFT*v*sEUT and 70%AFT*vs*EUT panels achieved the highest accuracy compared to the other panels for 100 (${r}^{2}$ = 0.967) and 200 (${r}^{2}$ = 0.984) SNPs, respectively, whereas 60%AFT*vs*EUT was the best performing panel with 300 (${r}^{2}$ = 0.988), 400 (${r}^{2}$ = 0.989) and 500 (${r}^{2}$ = 0.990) SNPs (Fig. 4b**)**. In Ethiopian crossbreds, the 70%AFT*vs*EUT was the best performing panel with 100 (${r}^{2}$ = 0.972) and 500 (${r}^{2}$ = 0.991) SNPs. The 50%AFT*vs*EUT performed better than the other panels with 200 (${r}^{2}$ = 0.983) and 300 (${r}^{2}$ = 0.987) SNPs, whereas 60%AFT*vs*EUT was the best performing panel with 400 (${r}^{2}$= 0.989) SNPs (Fig. 4c). In Tanzanian crossbreds, the 80%AFT*vs*EUT was the best performing panel with 100 (${r}^{2}$ = 0.961) SNPs, whereas the 60%AFT*vs*EUT was the best performing panel with 300 (${r}^{2}$ = 0.985) and 400 (*r*^*2*^ = 0.986) SNPs. The 50%AFT*vs*EUT performed better than the other panels with 200 (${r}^{2}$ = 0.978) and 500 (${r}^{2}$ = 0.989) SNPs (Fig. 4d). In Senegalese crossbreds, the 70%AFT*vs*EUT was the best performing panel with 100 (${r}^{2}$ = 0.976), 400 (${r}^{2}$ = 0.983) and 500 (${r}^{2}$ = 0.985) SNPs, whereas the 90%AFT*vs*EUT and 80%AFT*vs*EUT were the best performing panels for 200 (${r}^{2}$ = 0.982) and 300 (${r}^{2}$ = 0.984) SNPs, respectively (Fig. 4e).

When selecting SNPs from the 713 k dataset, the 70%AFT*vs*EUT panel achieved the highest accuracies in most crossbred populations and for most panel sizes. Table 3 illustrates the difference in ${r}^{2}$ between the 70%AFT*vs*EUT and the best performing panels, which shows that its accuracy of prediction was always negligibly lower than the best performing panel.

**Table 3:** The difference in ${r}^{2}$ between the 70%AFT*vs*EUT and the best-performing SNP panels for the 713 k dataset

#SNPs	Kenya	Uganda	Ethiopia	Tanzania	Senegal
100	0.000	− 0.002	0.000	− 0.002	0.000
200	0.000	0.000	− 0.001	− 0.001	− 0.003
300	0.000	0.000	− 0.001	− 0.001	0.000
400	0.000	− 0.001	0.000	− 0.001	0.000
500	0.000	0.000	0.000	− 0.001	0.000
1000	0.000	− 0.001	0.000	0.000	− 0.001
1500	− 0.001	0.000	0.000	0.000	− 0.004

Although larger panels always included the markers of the smaller panels, the accuracy for some panels did not always increase smoothly with increasing panel sizes. These deviations from a smooth increase in accuracy with increasing panel sizes are likely due to residual clustering of loci in LD as the interval between loci becomes smaller with increasing panel size.

To illustrate the effects of pruning on SNP selection, the distribution across the genome of the 1500 SNPs in the AllWestind*vs*EUT panel, with and without pruning, when selected from the 38 k dataset is shown in Additional file 1: Figure S1, and similarly for the 70%AFT*vs*EUT panel from the 713 k dataset in Additional file 2: Figure S2. The SNPs selected with pruning (see Additional file 1: Figure S1a and Additional file 2: Figure S2a) were evenly distributed across the genome compared to the SNPs selected without pruning (see Additional file 1: Figure S1b and Additional file 2: Figure S2b).

For both datasets, the results showed that small SNP panels performed better when a higher proportion of markers was selected to differentiate the African from European *Bos taurus* ancestral populations, compared to markers distinguishing *Bos indicus* from European *Bos taurus*. This reflects the relatively small genomic differences between African and European *Bos taurus* compared to the very large differences between European *Bos taurus* and *Bos indicus* [44–47], therefore requiring more markers for an accurate estimation of African and European *Bos taurus* proportions. For example, using public domain and new data that overlap with those in the current study, Gebrehiwot et al. [2] found that in a PCA of the SNP-based genomic relationship matrix, the second principal component (PC2) that separates African *Bos taurus* from European *Bos taurus* and *Bos indicus* explained 5.69% of the variance while PC1 that separates *Bos indicus* from European *Bos taurus,* explained 88.73% of the variance. In the same study, the breed differentiation between African and European *Bos taurus* ranged from ${F}_{\mathrm{ST}}$ = 0.211 to 0.332 compared with ${F}_{\mathrm{ST}}$ = 0.301 to 0.427 between European *Bos taurus* and *Bos indicus*, and ${F}_{\mathrm{ST}}$ = 0.372 to 0.492 between African *Bos taurus* and *Bos indicus* breeds. This reflects the more recent genetic divergence of the African and European *Bos taurus* groups compared with the divergence of *Bos taurus* and *Bos indicus* which is estimated to have occurred at least 200,000 years ago [44, 47–49].

### Selecting panels from 713 k versus 38 k SNP sets

The accuracy of estimated breed proportion was higher for all the panels selected from the 713 k compared to 38 k SNP set (see Additional file 3: Table S1). It was not possible to compare all the methods of SNP selection in all the populations because 713 k SNP genotype data were not available for all reference populations. In particular, the two populations of Senegalese crossbreds were genotyped either with the 38 k or the 713 k assay and could not be compared directly.

The most notable difference between the 38 k and 713 k datasets were the very small differences between most of the alternative panels in the 713 k dataset compared to the 38 k dataset, which means that the largest differences in accuracy between panels selected from the 713 k and 38 k datasets were found for the 10%AFT*vs*EUT panel (see Additional file 3: Table S1). The differences in accuracy between the two datasets were smallest for panels with greater weighting on markers that differentiated African from European *Bos taurus* breeds, which were also the optimum panels for both datasets. For example, the difference in accuracy between the 38 k and 713 k datasets for the 70%AFT*vs*EUT panel ranged from 0.005 to 0.027 for panels sizes between 300 and 500 SNPs and applied for the four countries included (see Additional file 3: Table S1).

In general, we expected that SNPs selected from a larger SNP set would provide a higher accuracy than that from a smaller SNP set because more SNPs of the desired characteristics should be present in the larger SNP set. According to Wang and Nielsen [50] and as illustrated here in Fig. 1, the BovineSNP50 BeadChip, which is the source of our 38 k dataset, is affected by substantial ascertainment bias, which results in many SNPs with a low MAF in *Bos indicus* breeds. This bias is an advantage for our selection of SNPs because it increases the number of markers with high discrimination power (i.e. large absolute difference in allele frequency between *Bos indicus* and *Bos taurus* populations) compared to an assay of similar size with lower bias. However, when selecting from the much larger number of SNPs in the 713 k data, there was remarkably little difference in accuracy between panels ranging from 30%AFT*vs*EUT to 90%AFT*vs*EUT, presumably because the much larger set of SNPs includes more SNPs with differences in extreme frequency between *Bos indicus* and European *Bos taurus* and also between African and European *Bos taurus*. This means that fewer SNPs are required to identify all three combinations of ancestry, and hence it matters less what proportion of SNPs is chosen from *Bos indicus versus* European *Bos taurus* and African *versus* European *Bos taurus*.

While the results show high accuracies of estimation in terms of ${r}^{2}$, it is interesting to know the standard errors (s.e.) of the estimates of breed composition. For the Kenyan crossbred data, which is the largest dataset, the between-animal standard deviation of the estimates of exotic breed proportion from the 713 k dataset was 0.214, and the s.e. of the estimates of exotic breed proportion were 0.005, 0.004, 0.003, 0.003 and 0.003 for the best panels of 100, 200, 300, 400 and 500 SNPs, respectively. For the best performing panel overall (70%AFT*vs*EUT), the achieved accuracy was lowest for the 100-SNP panel in Tanzania, which had an s.e. of 0.009 for the estimates of exotic ancestry.

Based on our results, we recommend the 70%AFT*vs*EUT panels selected from the 713 k SNP data for prediction of breed proportion in crossbred cattle across Africa. The gain in accuracy compared to optimum panels selected from the 38 k SNP data was small for the range of panel sizes of 300 or more SNPs that would likely be used in practice. However, the small difference in accuracy between a wide range of panels selected from the 713 k SNP data means that the 713 k SNP panels will be more robust to sampling errors of allele frequencies from the reference ancestral breeds. Thus, if new markers need to be chosen in other situations, the additional costs of genotyping populations with the 777 k SNP assay instead of the 50 k assay can be justified to ensure that the chosen set of markers will have a high accuracy in all situations.

### Parentage assignment

**Fig. 5:**
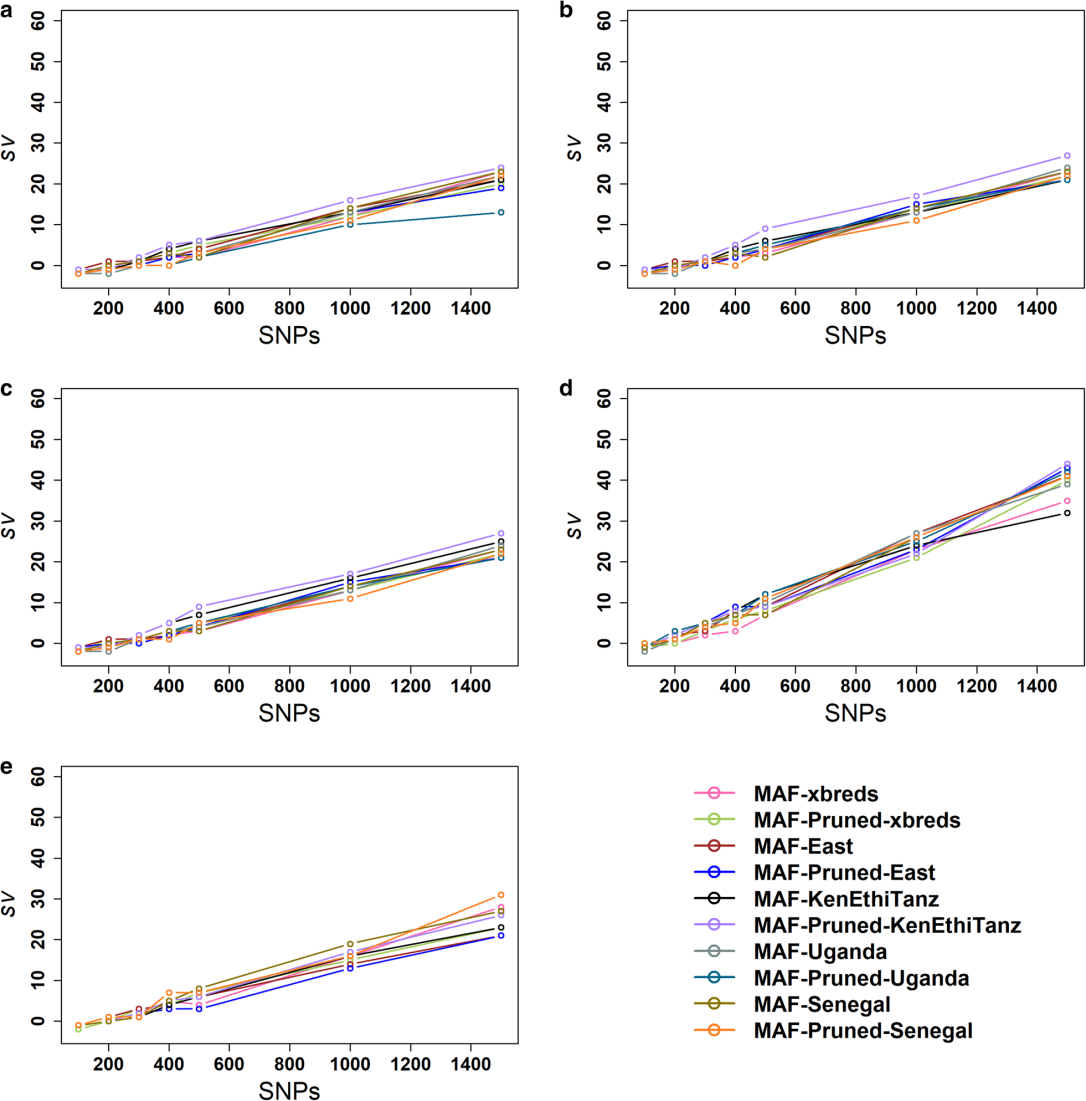
Separation values ($sv$) of parentage assignment for small SNP panels for Scenario 1 in **a** all African crossbreds **b** all East African crossbreds **c** Kenya-Ethiopia-Tanzania together **d** Ugandan **e** Senegal crossbreds

The $sv$ was used to compare the ability of different panels to identify parent–offspring pairs. The $sv$ in African populations for different panel sizes in Scenario 1, in which parents were selected among all the animals with known genotypes, are shown in Fig. 5. In all the African populations, the $sv$ was either negative or zero for the 100-SNP panels, whereas it was positive for a few of the 200-SNP panels. The $sv$ was positive and started to increase for all panels of 300 or more SNPs in Senegalese and Ugandan crossbred populations. However, the $sv$ value was still zero for a few panels for the groups of all crossbreds, all East African crossbreds, and Kenya-Ethiopia-Tanzania crossbreds. Our results show that an unambiguous parent–offspring assignment is not possible in all populations for all panels of 300 or less SNPs. Strucken et al. [15, 39] reported a similar finding for East African and East Asian cattle populations. Across all African crossbreds, the $sv$ became positive with 500 or more SNPs. Higher $sv$ values with 200 and 300 SNPs were obtained in Ugandan and Senegalese crossbreds compared to the other groups, which might be due to the higher genetic diversity of these populations.

To assess the impact of pruning on parentage assignments, the $sv$ value obtained from pruned and unpruned panels were compared for all panels of 200 to 500 SNPs in all crossbred groups. Generally, unlike the SNP panels selected for estimating the total dairy breed proportion, pruning did not show an obvious or consistent difference in accuracy of parentage assignment. For example, the pruned MAF-xbreds panel had a higher $sv$ value than the unpruned MAF-xbreds with 300 SNPs for Ugandan (Fig. 5d) and Senegalese (Fig. 5e) crossbreds, whereas the unpruned MAF-xbreds panel performed better from 300 to 500 SNPs in groups of all crossbreds (Fig. 5a), all East African (Fig. 5b) and Kenya-Ethiopia-Tanzania (Fig. 5c) crossbreds, and with 400 and 500 SNPs for Ugandan crossbreds. Likewise, the unpruned MAF-East panel achieved a higher $sv$ value than the pruned MAF-East with 300 SNPs for Ugandan crossbreds, while both pruned and unpruned panels performed equally with 300 SNPs for the group of all crossbreds. However, the unpruned MAF-East panel had a higher $sv$ value than the pruned MAF-East panel with 200 SNPs for all crossbred groups, except for Ugandan crossbreds, and with 300 SNPs for all East, Kenya-Ethiopia-Tanzania, and Senegalese crossbreds.

The $sv$ in Scenario 2, where it was assumed that it was known in advance which animals were parents, was either negative or zero with 100 SNPs, while it was positive for the majority of panels for 200 SNPs in all groups (see Additional file 4: Figure S3). With 300 SNPs, only the MAF-Uganda panel in the all African crossbred group had a negative $sv$. For all the panels and all the populations, the $sv$ in Scenario 2 were often substantially larger than the $sv$ in Scenario 1. This is expected given the much smaller search space to assign parents to offspring in Scenario 2, which leads to a much larger range in the number of opposing homozygotes observed for false parent–offspring pairs in Scenario 1. For example, for the group of all African crossbreds, in which there are 301 parents and 2892 potential progeny, there are 5,096,028 possible parent–offspring pairs to be tested in Scenario 1 *versus* 870,492 in Scenario 2.

### Power of assignment and power of exclusion

**Fig. 6:**
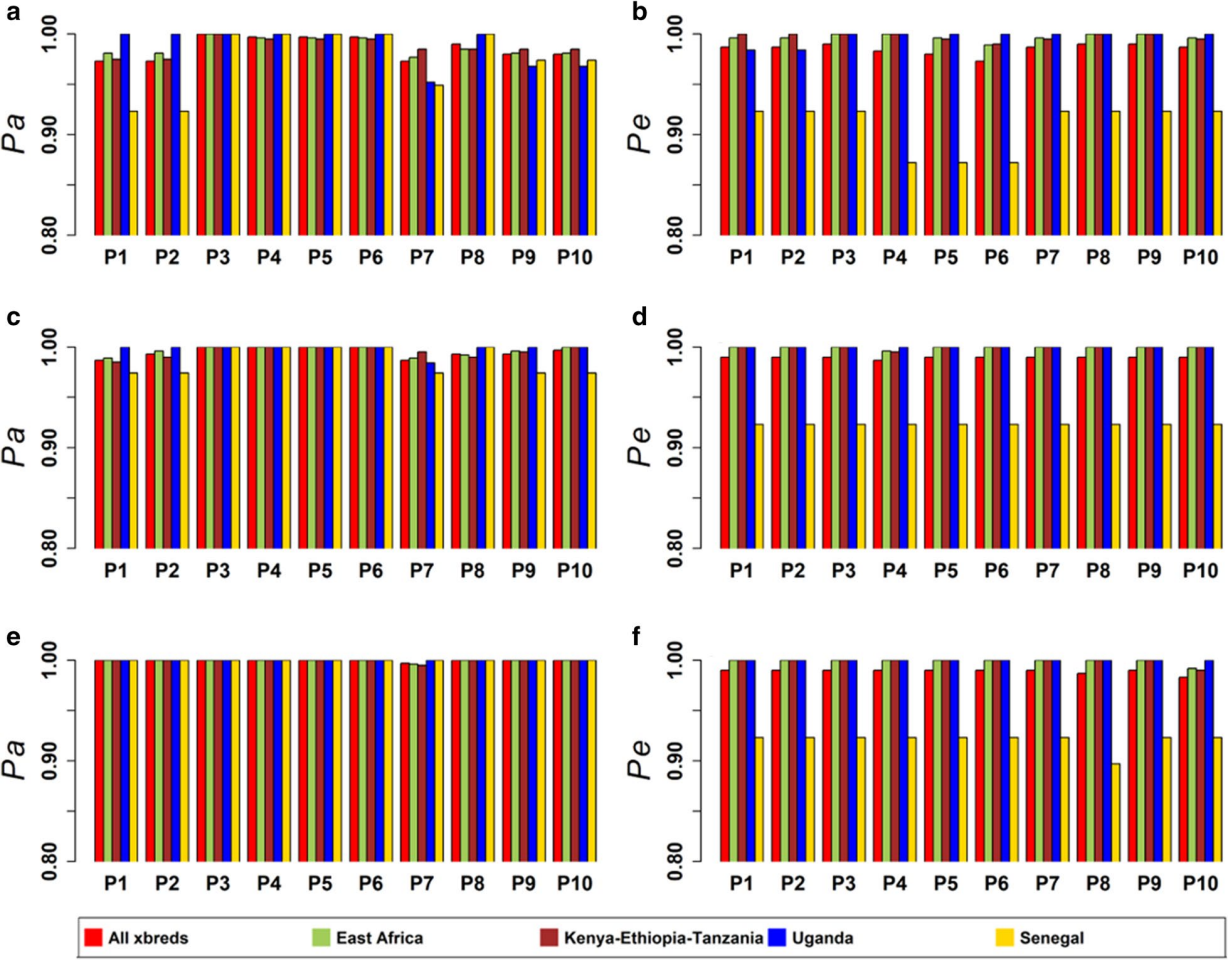
Power of assignment ($Pa$) and power of exclusion ($Pe$) of 200 SNP (**a**, **b**), 300 SNP (**c**, **d**), and 400 SNP (**e**, **f**) panels. P1 = MAF-xbreds, P2 = MAF-Pruned-xbreds, P3 = MAF-East, P4 = MAF-Pruned-East, P5 = MAF-KenEthTan, P6 = MAF-Pruned-KenEthTan, P7 = MAF-Uganda, P8 = MAF-Pruned-Uganda, P9 = MAF-Senegal, and P10 = MAF-Pruned-Senegal

The powers of assignment ($Pa$) and exclusion ($Pe$) for panels of 200, 300 and 400 SNPs are shown in Fig. 6. $Pe$ were generally slightly lower than $Pa$. Only the MAF-East panel achieved a $Pa$ of 1 in all groups of crossbreds with 200 SNPs, whereas the other panels had a $Pa$ of 1 with 300 and 400 SNPs. In contrast, no panel of 200 or more SNPs achieved a $Pe$ of 1 across all crossbred groups. According to McClure et al. [42], increasing the number of markers in a panel to at least 500 SNPs achieves a higher $Pe$. However, a larger number of markers usually leads to an increased cost of genotyping, thus, there is a trade-off between accuracy and cost to be optimized. Still, in the present case, if the cost favors a panel as small as possible, the MAF-East panel would be preferred for parentage assignment in African crossbred cattle because for panels of 200 or more SNPs, it achieved a $Pa$ of 1 in all crossbred groups and a $Pe$ of 1 in the majority of the populations. This panel also achieved $sv$ greater than 0 in all populations.

A problem that is often associated with the use of array-based SNPs in population studies is ascertainment bias in the determination of which SNPs are selected for an assay [51, 52]. In the present case, when selecting SNPs from the 38 k data, it was possible to find SNPs that had a high MAF and high accuracy of assigning parent–offspring pairs in all populations. Therefore, obviously any ascertainment bias present on the original 50 k SNP assay has not limited the current application.

### Performance of East African reference panels in West African crossbreds

Not all markers selected by Strucken et al. [15] for prediction of breed proportion in East African crossbred cattle were available in our 713 k data from Senegal. The numbers of SNPs found in the Senegal data that were also found in the reference panels were 97, 196, 295, 391, 490, 981, and 1470 SNPs for the original panel sizes of 100, 200, 300, 400, 500, 1000 and 1500, respectively.

**Fig. 7:**
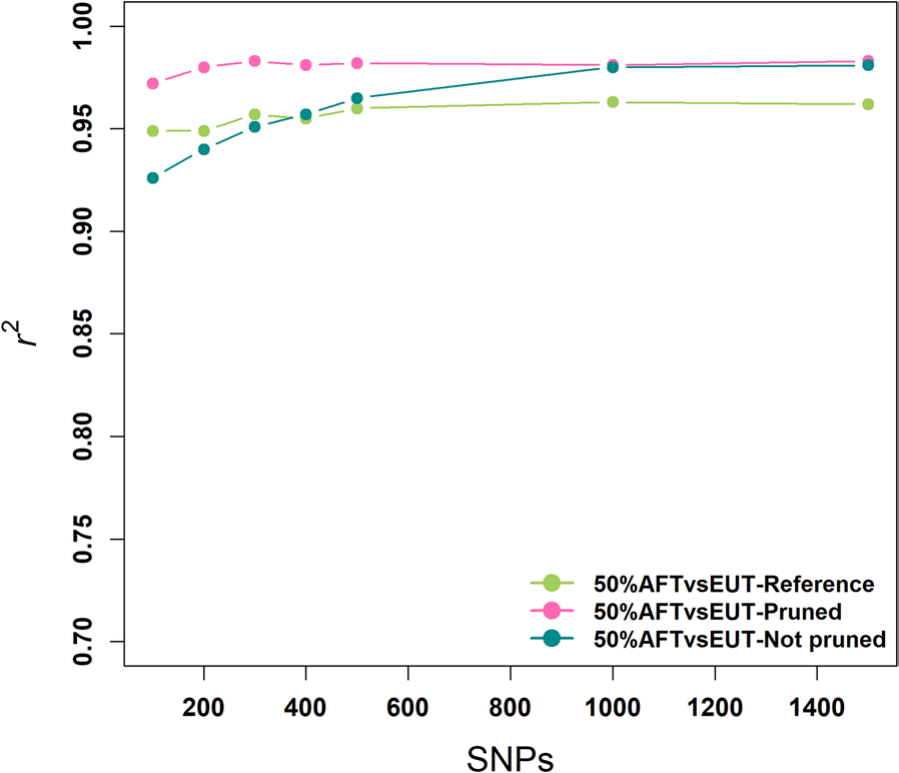
Performance of a reference panel (Strucken et al. [15]) and the newly derived panels (pruned and not-pruned) for estimating dairy breed proportions in a Senegalese crossbred population

The best panel for prediction of dairy breed proportion by Strucken et al. [15] (NelNdEU) achieved an accuracy higher than 0.96 with 100 markers in East African crossbred populations. The achieved accuracies flattened at about 500 markers with an ${r}^{2}$ higher than 0.98. The same panel achieved a somewhat lower ${r}^{2}$ in the Senegalese crossbreds with 100 markers and accuracies only marginally increased with more markers in the West African population (Fig. 7).

To make a direct comparison with the reference panel (NelNdEU) recommended by Strucken et al. [15], panels of 100 to 1500 SNPs for the 50%AFT*vs*EUT panel were selected from the 713 k SNP data without pruning. Figure 7 compares the accuracy of this panel with the reference panel in the Senegalese crossbred population. In spite of the slightly smaller number of SNPs in the reference panel, the accuracies of the reference panels were higher than those of the unpruned 50%AFT*vs*EUT panel for up to 300 SNPs. However, with more SNPs, especially with 1000 and 1500 SNPs, the 50%AFT*vs*EUT panel was more accurate than the reference panel.

Figure 7 also shows the accuracy of the 50%AFT*vs*EUT panels selected with pruning, which also appears in Fig. 4. The performance of the pruned 50%AFT*vs*EUT panel was always higher than the reference and the unpruned 50%AFT*vs*EUT panels. As noted in the Methods section and illustrated in Additional file 1: Figure S1 and Additional file 2: Figure S2, the unpruned selection of SNPs leads to clustering of SNPs across the genome, which will lead to high LD in crossbred populations. He et al. [53] also showed that reducing the LD by pruning selected marker panels allowed the use of a smaller number of markers for the estimation of breed proportion in cattle. Frkonja et al. [54] observed that higher accuracy is attained when the SNPs for the estimation of breed proportions are distributed across the genome rather than representing a sub-set of chromosomes.

Comparison of the performance of the optimum panel designed by Strucken et al. [15] with that of the best performing panel in this study (70%AFT*vs*EUT) showed similar accuracies for the East African crossbred populations, but a lower accuracy of the reference panel for the West African crossbred populations. Our analysis included a pool of several populations of *Bos indicus* and African *Bos taurus* reference breeds, whereas Strucken et al. [15] only used Nellore and N’Dama as *Bos indicus* and African *Bos taurus*, respectively, which might have been less representative of the genetic diversity in crossbred cattle from different parts of Africa, and their smaller sample sizes would result in lower accuracy of the allele frequency estimates. In addition, the improved method of SNP selection developed here generates more accurate panels than the previous method, particularly for small panel sizes.

We were unable to determine the accuracy of the parentage panels obtained by Strucken et al. [15] for our West African crossbred population because there were only four parent–offspring pairs in the 713 k data for Senegal, and we deemed that this was insufficient to obtain meaningful estimates of the $sv$, $Pa$ and $Pe$ values.

In human populations, the portability of the ancestry-informative SNPs depends on the relationship between the populations examined [55]. In the case of African crossbred cattle, when selecting SNPs from 713 k SNP data, optimum panels can be found that work well across all populations, notwithstanding the very large genetic differences between their African indigenous breed ancestries. Given the large genetic differences in indigenous breeds between East and West African cattle, the high accuracy of the optimum panels developed here is likely to apply in all African dairy cattle populations, which are crosses between indigenous and exotic dairy breeds. The results for parentage testing panels look equally promising; however, we had an insufficient number of animals to derive and test panels from 713 k data in both East and West African samples. Although the optimum panels should work well in all crossbred populations, it is possible that even more accurate panels could be found if panels were derived from and tested in 713 k SNP data.

## Conclusions

When more than two breed ancestries need to be estimated with small SNP panels, the choice of the SNPs should place the greatest weighting on SNPs that differentiate the most closely related ancestral populations. This is particularly important when the set of SNPs from which SNP panels can be selected is relatively small (38 k *versus* 713 k in our comparison). A single panel of 300 to 500 SNPs will provide high accuracy of the estimation of exotic dairy proportion in West and East African crossbred cattle populations, and given the shared breed ancestral background we expect it will have high accuracy in all other crossbred populations in Africa. We propose a marker panel with a minimum of 200 SNPs for the estimation of breed proportion (70%AFT*vs*EUT) and for parentage assignment (MAF-East). Rapid and cheap prediction of total dairy breed proportion and parentage verification in African crossbred cattle populations will allow optimization of crossbreeding and on-going genetic improvement in most of the situations where pedigree information is incomplete or unavailable.

## Supplementary Information

**Additional file 1: Figure S1**. Physical genome position (Mb) of 1500 SNPs of the AllWestind*vs*EUT panel (a) pruned (b) unpruned. The file provided shows the physical position of SNPs selected from 38k SNPs present on the Illumina BovineSNP50v2 and BovineHD Beadchip (Illumina Inc., San Diego, USA).

**Additional file 2: Figure S2.** Physical genome position (Mb) of 1500 SNPs of the 70%AFT*vs*EUT panel (a) pruned (b) unpruned. The file provided shows the physical position of SNPs selected from 713k SNPs present on the Illumina BovineHD Beadchip (Illumina Inc., San Diego, USA).

**Additional file 3: Table S1. **Difference in ${r}^{2}$ for dairy breed proportion estimation between panels selected from 713k and 38k SNP datasets. The provided file shows a table with the difference in accuracy (*r*^2^) of dairy breed proportions estimated for nine SNP panels selected from 713k and 38k SNPs present on the Illumina BovineSNP50v2 and BovineHD Beadchip (Illumina Inc., San Diego, USA). Accuracy differences are given for seven different panel sizes ranging from 100 to 1500 SNPs and separated out for crossbred populations samples in Kenya, Uganda, Ethiopia, and Tanzania.

**Additional file 4: Figure S3.** Parentage assignment using the separation value ($sv$*)* for small SNP panels for Scenario 2 in (a) all African crossbreds, (b) all East African crossbreds, (c) in Kenya-Ethiopia-Tanzania together, (d) in Ugandan, and (e) in Senegalese crossbreds. The provided file shows the accuracy of parentage assignments based on the separation value in five different crossbred populations of African dairy cattle. Ten SNP panels selected from 713k and 38k SNPs present on the Illumina BovineSNP50v2 and BovineHD Beadchip (Illumina Inc., San Diego, USA), were tested using seven different panel sizes ranging from 100 to 1500 SNPs.

## Data Availability

Data were sourced from a variety of public domain and privately held databases as detailed in the paper. In most cases, the data held privately is available on request to the institution owning the data.
