# Supplementary figures and images for: SNP panels for the estimation of dairy breed proportion and parentage assignment in African crossbred dairy cattle
Source: Genet Sel Evol. 2021 Mar 2;53:21. doi: 10.1186/s12711-021-00615-4 (PMC7923343; doi:10.1186/s12711-021-00615-4)

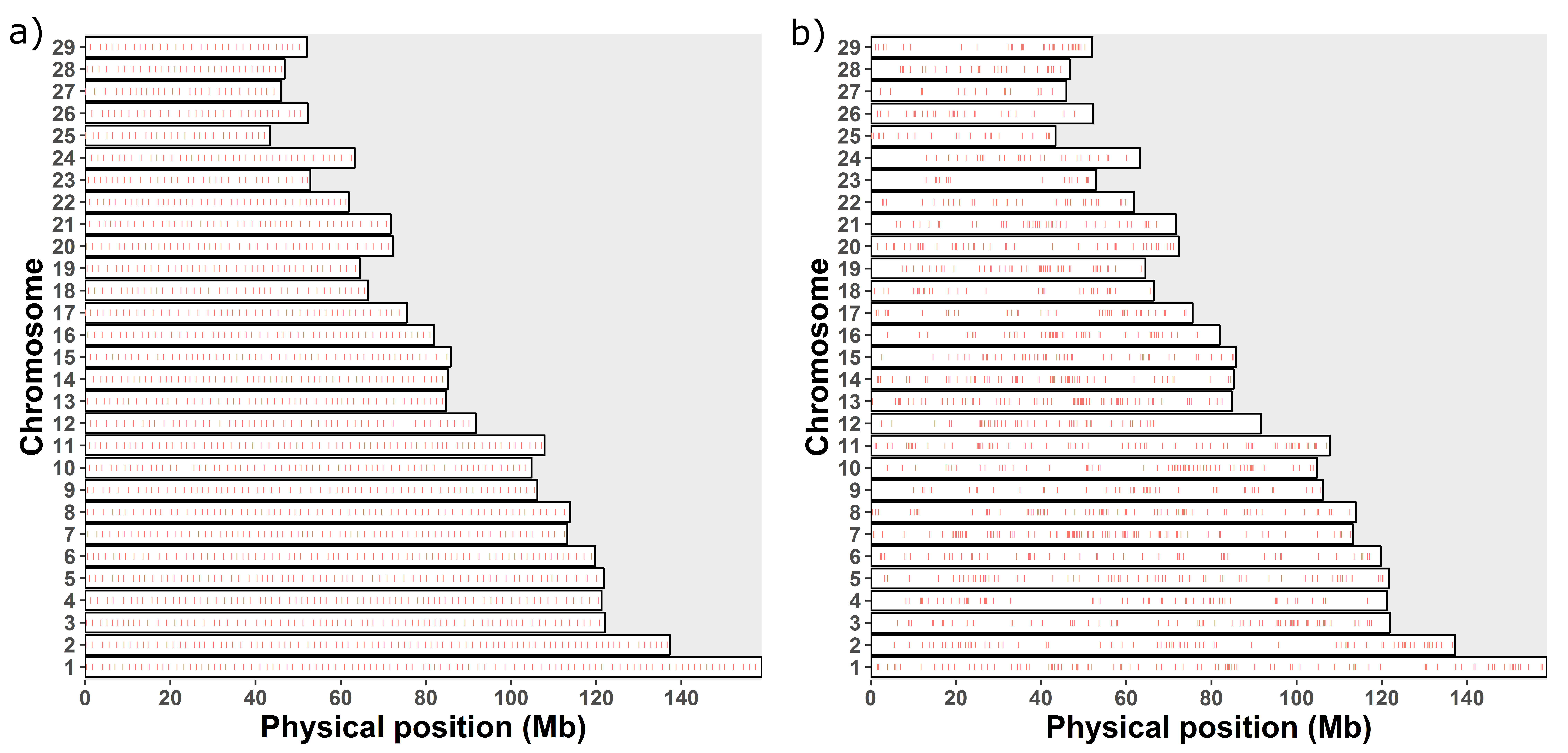

Supplement: Supplementary file 1 — Additional file 1: Figure S1. Physical genome position (Mb) of 1500 SNPs of the AllWestindvsEUT panel (a) pruned (b) unpruned. The file provided shows the physical position of SNPs selected from 38k SNPs present on the Illumina BovineSNP50v2 and BovineHD Beadchip (Illumina Inc., San Diego, USA). [file 12711_2021_615_MOESM1_ESM.tiff]

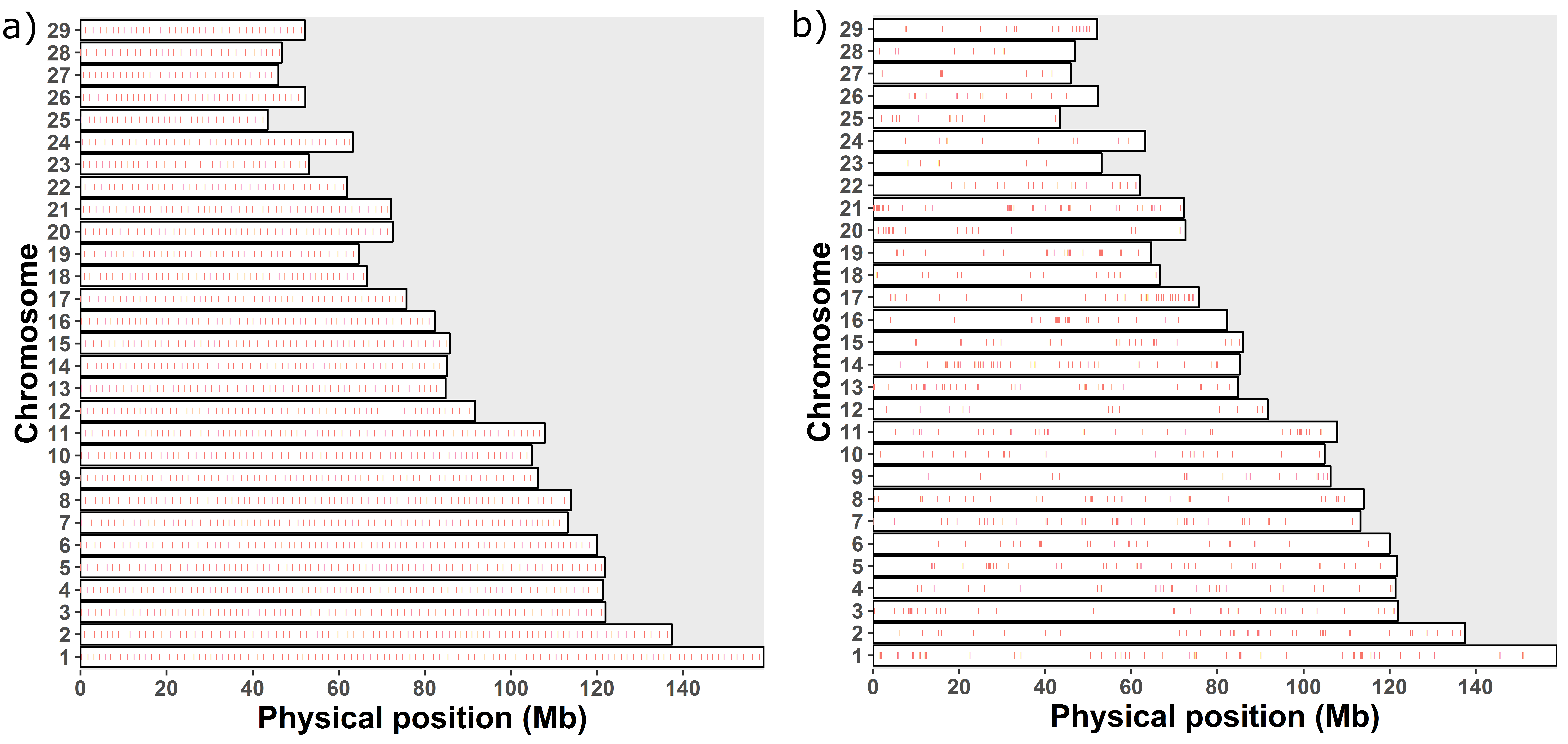

Supplement: Supplementary file 2 — Additional file 2: Figure S2. Physical genome position (Mb) of 1500 SNPs of the 70%AFTvsEUT panel (a) pruned (b) unpruned. The file provided shows the physical position of SNPs selected from 713k SNPs present on the Illumina BovineHD Beadchip (Illumina Inc., San Diego, USA). [file 12711_2021_615_MOESM2_ESM.tiff]

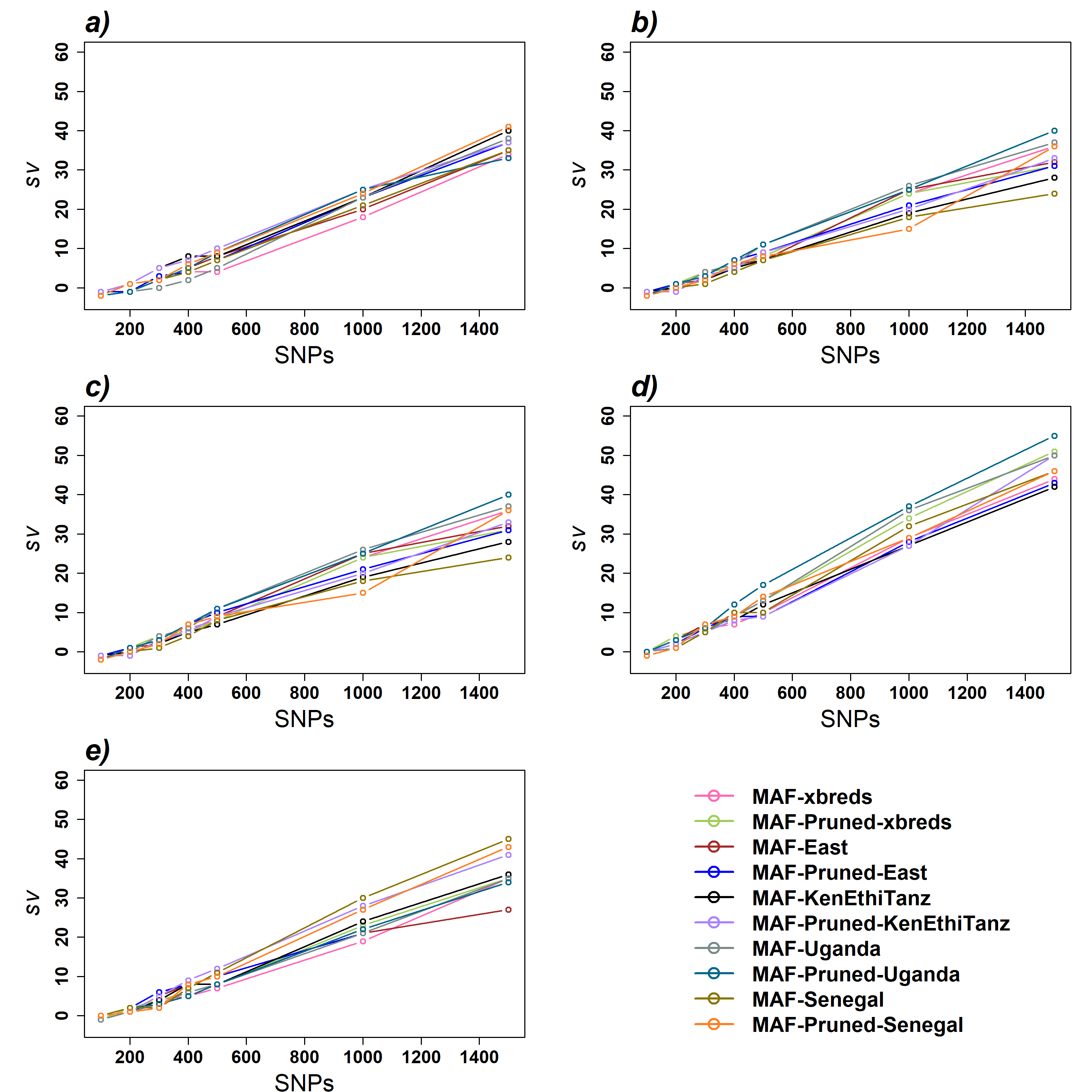

Supplement: Supplementary file 4 — Additional file 4: Figure S3. Parentage assignment using the separation value ($sv$sv) for small SNP panels for Scenario 2 in (a) all African crossbreds, (b) all East African crossbreds, (c) in Kenya-Ethiopia-Tanzania together, (d) in Ugandan, and (e) in Senegalese crossbreds. The provided file shows the accuracy of parentage assignments based on the separation value in five different crossbred populations of African dairy cattle. Ten SNP panels selected from 713k and 38k SNPs present on the Illumina BovineSNP50v2 and BovineHD Beadchip (Illumina Inc., San Diego, USA), were tested using seven different panel sizes ranging from 100 to 1500 SNPs. [file 12711_2021_615_MOESM4_ESM.tiff]
