# Supplementary material for: SNP panels for the estimation of dairy breed proportion and parentage assignment in African crossbred dairy cattle
Source: Genet Sel Evol. 2021 Mar 2;53:21. doi: 10.1186/s12711-021-00615-4 (PMC7923343; doi:10.1186/s12711-021-00615-4)
Supplement: Supplementary file 3 — Additional file 3: Table S1. Difference in ${r}^{2}$r2 for dairy breed proportion estimation between panels selected from 713k and 38k SNP datasets. The provided file shows a table with the difference in accuracy (r2) of dairy breed proportions estimated for nine SNP panels selected from 713k and 38k SNPs present on the Illumina BovineSNP50v2 and BovineHD Beadchip (Illumina Inc., San Diego, USA). Accuracy differences are given for seven different panel sizes ranging from 100 to 1500 SNPs and separated out for crossbred populations samples in Kenya, Uganda, Ethiopia, and Tanzania. [file 12711_2021_615_MOESM3_ESM.docx]

| Crossbreds | #SNPs | Panel 1 | Panel 2 | Panel 3 | Panel 4 | Panel 5 | Panel 6 | Panel 7 | Panel 8 | Panel 9 |
| --- | --- | --- | --- | --- | --- | --- | --- | --- | --- | --- |
| Kenya | 100 | 0.193 | 0.190 | 0.087 | 0.075 | 0.057 | 0.054 | 0.042 | 0.037 | 0.041 |
| Kenya | 200 | 0.118 | 0.080 | 0.051 | 0.044 | 0.034 | 0.028 | 0.029 | 0.031 | 0.027 |
| Kenya | 300 | 0.076 | 0.041 | 0.031 | 0.026 | 0.023 | 0.021 | 0.019 | 0.020 | 0.024 |
| Kenya | 400 | 0.055 | 0.030 | 0.023 | 0.017 | 0.020 | 0.015 | 0.013 | 0.014 | 0.013 |
| Kenya | 500 | 0.050 | 0.033 | 0.013 | 0.012 | 0.016 | 0.013 | 0.005 | 0.013 | 0.011 |
| Kenya | 1000 | 0.014 | 0.010 | 0.011 | 0.009 | 0.009 | 0.009 | 0.004 | 0.009 | 0.005 |
| Kenya | 1500 | 0.006 | 0.006 | 0.006 | 0.007 | 0.006 | 0.006 | 0.065 | 0.004 | 0.004 |
| Uganda | 100 | 0.230 | 0.208 | 0.086 | 0.071 | 0.064 | 0.059 | 0.052 | 0.050 | 0.041 |
| Uganda | 200 | 0.126 | 0.077 | 0.050 | 0.046 | 0.033 | 0.031 | 0.033 | 0.031 | 0.031 |
| Uganda | 300 | 0.067 | 0.038 | 0.030 | 0.028 | 0.022 | 0.020 | 0.023 | 0.022 | 0.029 |
| Uganda | 400 | 0.061 | 0.029 | 0.029 | 0.015 | 0.021 | 0.016 | 0.014 | 0.018 | 0.015 |
| Uganda | 500 | 0.045 | 0.036 | 0.014 | 0.013 | 0.016 | 0.016 | 0.006 | 0.014 | 0.010 |
| Uganda | 1000 | 0.016 | 0.010 | 0.014 | 0.012 | 0.011 | 0.010 | 0.004 | 0.010 | 0.005 |
| Uganda | 1500 | 0.006 | 0.010 | 0.007 | 0.010 | 0.008 | 0.008 | 0.078 | 0.004 | 0.004 |
| Ethiopia | 100 | 0.230 | 0.210 | 0.103 | 0.090 | 0.065 | 0.064 | 0.053 | 0.048 | 0.040 |
| Ethiopia | 200 | 0.149 | 0.086 | 0.060 | 0.047 | 0.033 | 0.028 | 0.025 | 0.031 | 0.023 |
| Ethiopia | 300 | 0.084 | 0.039 | 0.031 | 0.030 | 0.021 | 0.019 | 0.021 | 0.020 | 0.022 |
| Ethiopia | 400 | 0.064 | 0.037 | 0.021 | 0.018 | 0.023 | 0.018 | 0.011 | 0.015 | 0.014 |
| Ethiopia | 500 | 0.051 | 0.036 | 0.015 | 0.012 | 0.014 | 0.016 | 0.006 | 0.013 | 0.013 |
| Ethiopia | 1000 | 0.014 | 0.012 | 0.010 | 0.008 | 0.007 | 0.007 | 0.005 | 0.009 | 0.008 |
| Ethiopia | 1500 | 0.005 | 0.007 | 0.006 | 0.007 | 0.006 | 0.005 | 0.071 | 0.005 | 0.005 |
| Tanzania | 100 | 0.211 | 0.241 | 0.101 | 0.076 | 0.054 | 0.049 | 0.048 | 0.044 | 0.040 |
| Tanzania | 200 | 0.154 | 0.092 | 0.067 | 0.048 | 0.037 | 0.026 | 0.024 | 0.028 | 0.025 |
| Tanzania | 300 | 0.090 | 0.049 | 0.032 | 0.031 | 0.027 | 0.029 | 0.027 | 0.024 | 0.026 |
| Tanzania | 400 | 0.076 | 0.036 | 0.022 | 0.023 | 0.025 | 0.023 | 0.016 | 0.022 | 0.017 |
| Tanzania | 500 | 0.068 | 0.034 | 0.018 | 0.019 | 0.021 | 0.023 | 0.009 | 0.018 | 0.014 |
| Tanzania | 1000 | 0.014 | 0.018 | 0.018 | 0.013 | 0.015 | 0.015 | 0.008 | 0.013 | 0.010 |
| Tanzania | 1500 | 0.006 | 0.012 | 0.010 | 0.014 | 0.011 | 0.011 | 0.078 | 0.007 | 0.008 |

**Table S1**. The difference in *r^2^* between panels selected from 713k and 38k SNP datasets.

Panel 1 = 10%AFT*vs*EUT, Panel 2 = 20%AFT*vs*EUT, Panel 3 = 30%AFT*vs*EUT, Panel 4 = 40%AFT*vs*EUT, Panel 5 = 50%AFT*vs*EUT, Panel 6 = 60%AFT*vs*EUT, Panel 7 =70%AFT*vs*EUT, Panel 8 = 80%AFT*vs*EUT, Panel 9 = 90%AFT*vs*EUT.
